# Postpartum Depression, Catatonia and Covid-19 Infection: One Case, Different Clinical Presentations

**DOI:** 10.1192/j.eurpsy.2022.1450

**Published:** 2022-09-01

**Authors:** D. Leite, A.F. Antunes

**Affiliations:** Hospital Espirito Santo de Évora, Departamento De Psiquiatria E Saúde Mental, Évora, Portugal

**Keywords:** postpartum depression, Covid-19, Catatonia

## Abstract

**Introduction:**

Post-partum depression may occur in the first year after childbirth in approximately 25% of women, at times presenting with psychotic symptoms and catatonic states. Catatonia is a psychomotor syndrome that occurs in association with various neuropsychiatric disorders and can be described according to the characteristics of its manifestation in types such as retarded or agitated.

**Objectives:**

We report the case of a patient with postpartum depression and catatonic syndrome who, after a session of electroconvulsive therapy, was infected with Sars-COV-2, suspended treatment, and had her condition aggravated with distinct clinical manifestations.

**Methods:**

Clinical case report and non-systematic review of articles consulted in the PubMed platform.

**Results:**

A 24-year-old patient develops depressive symptoms and obsessive behaviour 6 months after delivery and deteriorates with mutism, stupor and motor immobility. She was hospitalised and medicated with lorazepam, with no improvement. One session of electroconvulsive therapy was carried out with improvement of the symptoms. Due to an inpatient Covid-19 outbreak, in which the patient was infected, treatment was suspended. During isolation, deterioration of the patient’s condition was observed with psychomotor agitation, bizarre behaviour, and perseverative speech. The patient resumed treatment with ECT, with total remission of the catatonic syndrome and improvement of the affective symptoms.

**Conclusions:**

Catatonic syndromes are relatively rare, but its association with post-partum depression is not so uncommon. The occurrence of different presentations of catatonia, although described as possible in the same episode in the literature, were not found in any clinical studies reviewed, which leads us to conclude that it is an uncommon situation.

**Disclosure:**

No significant relationships.

